# An Interpretable Digital Twin for Self-Aware Industrial Machines

**DOI:** 10.3390/s24010004

**Published:** 2023-12-19

**Authors:** João L. Vilar-Dias, Adelson Santos S. Junior, Fernando B. Lima-Neto

**Affiliations:** School of Computer Sciences, University of Pernambuco, Recife 50720-001, Brazil; assj2@ecomp.poli.br (A.S.S.J.); fbln@ecomp.poli.br (F.B.L.-N.)

**Keywords:** Digital Twin, self-awareness, Industry 4.0, artificial intelligence

## Abstract

This paper presents a proposed three-step methodology designed to enhance the performance and efficiency of industrial systems by integrating Digital Twins with particle swarm optimization (PSO) algorithms while prioritizing interpretability. Digital Twins are becoming increasingly prevalent due to their capability to offer a comprehensive virtual representation of physical systems, thus facilitating detailed simulations and optimizations. Concurrently, PSO has demonstrated its effectiveness for real-time parameter estimation, especially in identifying both standard and unknown components that influence the dynamics of a system. Our methodology, as exemplified through DC Motor and Hydraulic Actuator simulations, underscores the potential of Digital Twins to augment the self-awareness of industrial machines. The results indicate that our approach can proficiently optimize system parameters in real-time and unveil previously unknown components, thereby enhancing the adaptive capacities of the Digital Twin. While the reliance on accurate data to develop Digital Twin models is a notable consideration, the proposed methodology serves as a promising framework for advancing the efficiency of industrial applications. It further extends its relevance to fault detection and system control. Central to our approach is the emphasis on interpretability, ensuring a more transparent understanding and effective usability of such systems.

## 1. Introduction

The industrial landscape has dramatically transformed, evolving from manual operations to sophisticated, automated systems [1]. Since the third industrial revolution, humanity has made significant efforts on the manipulation of the manufacturing process to obtain desired outputs, i.e., the improved control of the executed processes. The effective control of dynamic systems, which are characterized by changes in their states over time requires a thorough understanding of their dynamics [2]. The modern and classic control theory has an important focus on studies of these systems and their behavior, building models of the physical systems and using them for the development of system controllers.

However, real-world systems often present significant challenges due to unpredictable and sometimes unknown factors, such as aggressive environments, weather conditions, variations in operating conditions, deterioration, aging, and other factors [3]. That is, their operation and behavior may vary over time, due to external factors and physical changes in their components and materials. This may result in failures in the control or operation, causing productivity, financial, or even personal losses to all involved.

Adaptive models and adaptive control are used to enhance the controllers’ adaptability to environmental and operational conditions [4]. Nonetheless, due to its complexity and uncertainties, it may need special instrumentation besides human expertise for development. Moreover, industrial applications often require a better interpretation and understanding of the model mechanism. The decisions made based on these model predictions may have a significant impact on safety, production, and financial among other critical factors [3].

To address these challenges, Digital Twins have emerged as a promising technology for industrial applications [5,6,7,8]. These Digital Twins act as advanced models; they can provide a virtual representation of the physical systems to be used for different purposes, such as simulation and behavior prediction, control, and maintenance [9]. In addition, a continuous update of the Digital Twins with real-time data allows for more accurate predictions and better decision-making outputs, increasing the machine’s self-awareness [8].

Self-aware computing, which is considered a promising area of study and may become the future of computational systems, focuses on systems capable of seeking and maintaining knowledge about themselves, as well as learning and reasoning about what is being done so that they can express themselves or achieve their goals [10,11,12]. In turn, self-aware systems can bring greater efficiency to industrial environments, increasing adaptability and reducing production downtime due to maintenance.

In recent years, many artificial intelligence techniques have been developed in order to perform optimizations in complex functions without the need for prior knowledge about them [13]. This type of technique is collectively called metaheuristics [14], a group that includes widely used algorithms such as genetic algorithms (GA) and particle swarm optimization (PSO) [15].

Such an ability to optimize unknown functions can be useful for machines to become self-aware in a reasoning process in which they try to find out what their current operating parameters are. In this context, techniques such as particle swarm optimization (PSO) further enhance this awareness by estimating meaningful model parameters, providing environmental and operational conditions information to the Digital Twin.

However, traditional approaches often lack interpretability and explainability, which can limit their practical applications. These limitations require specialized instrumentation and expertise to develop, making it challenging for non-experts to understand the model mechanism and interpret the results.

### 1.1. Digital Twins and Self-Awareness

Self-awareness could improve the machine’s behavior based on the environment and operation conditions in an adaptive way, perceiving abnormalities in its fundamental characteristics and acting accordingly, which can lead to increased productivity and efficiency, as well as reduced downtime. That must accompany the operation and process data as soon as they are available, making the virtual representation accurate and reliable even under adverse conditions, enabling self-adaptation and optimization of automatic process control in industrial environments.

Zhang et al. (2020) discuss a conceptual framework for self-aware Digital Twins, combining the conventional Digital Twin concept with the generic self-awareness architecture (stimulus, interaction, time, and goal awareness) as a way to improve intelligent planning and decision-making. Such discussion brings more emphasis for the necessity of more intelligent Digital Twins to improve efficiency in complex systems [16].

### 1.2. System Interpretability

In the industrial domain, the ability to interpret and understand system models is crucial for safety, efficiency, and innovation [5]. The ones making the decisions regarding system operation, for example, prefer to agree to something that can be understood at a certain level than to some black-box model telling them what they need to do to increase efficiency, productivity, etc. Hence, it is important that the Digital Twin could provide an interpretable system representation.

White box representations or models based on the mechanical and electronic physics of the real system could provide such an interpretation. One can relate specific parts of the model structure and its parameters to the different components of the real system, whose known characteristics and parameters can be monitored by sensors.

Nonetheless, the machines’ parameters’ values could be difficult to obtain from real machines’ instrumentation or manufacturers’ information. This could be due to diverse factors including unavailability of precise data, high costs of sensor instrumentation, complexity of the dynamic system and interaction between different components of the system [17]. Hence, estimation techniques for the non-instrumented but known parameters are necessary.

### 1.3. System Adaptability

In light of these considerations, continuous or online parameters estimation allows to discover non-instrumented parameters values and adapts the models during the system operation using real-time data. This, in such a way that its behavior represents the real system properly [17]. It can be addressed as an optimization problem, where the cost function is minimized to guarantee the model adaptation [18].

Due to the increased complexity of real systems, which are reflected in the number of parameters or even in processing large amounts of data, Swarm Intelligence, e.g., PSO, stands out among optimization techniques for performing a search for the solution in a decentralized manner, with processing divided between individuals, exploring simple characteristics and behaviors for the synergistic generation of information and knowledge [15].

Nevertheless, the Digital Twin approach may face limitations in capturing all the variables and parameters that influence the system outputs. These variables may not have been present at the time of the model creation or may not have been sufficiently captured in the data used to generate the model. Additionally, failures or unexpected changes in the system may lead to a divergence between the Digital Twin’s predictions and the real system outcomes.

Hence, the Digital Twin model needs to take into account the instrumented known parameters, non-instrumented known parameters, and unknown components and parameters that could better explain the system’s behavior within a certain operational time frame.

### 1.4. Objective and Organization

While dynamic systems offer immense potential, the challenges of model interpretability and explainability often hinder their full realization. These limitations require specialized instrumentation and expertise to develop, making it challenging for non-experts to understand the model mechanism and interpret the results.

This work has four main research goals:

1. Enhancing the interpretability and adaptability of Digital Twins;
2. Proposing a hybrid methodology for creating interpretable Digital Twins with online parameter estimation and unknown components discovery;
3. Providing a practical framework for augmenting self-awareness and decision-making in industrial machinery;
4. Executing the empirical validation of the interpretability and adaptability of the Digital Twins approach in two study cases of common industrial machines.

The primary objective of this work is to propose a hybrid methodology for creating an interpretable Digital Twin that synergizes online parameter estimation with unknown components using particle swarm optimization, then, enhancing the interpretability and adaptability of Digital Twins. The devised approach addresses the dual challenges of interpretability and adaptability inherent in Digital Twins and provides a practical framework for augmenting self-awareness and decision-making in industrial machinery. For the empirical validation of this approach, an industrial DC motor and a hydraulic actuator were selected as the case study.

This paper is organized as follows. A background of the Digital Twins application and the use of the PSO algorithm for parameter estimation and unknown components discovery is depicted in Section 2. Section 3 presents the proposed methodology. Section 4 presents a case study. Section 5 discusses the simulation and results. Finally, Section 6 summarizes the conclusions.

## 2. Background

### 2.1. Digital Twins Applications

The use of Digital Twins has been gaining popularity in various industries due to their ability to accurately replicate real-world systems. Aerospace, manufacturing, and robotics are notable examples where this technology has made a significant impact. For instance, in the aerospace industry, Digital Twins aid in the predictive maintenance and real-time monitoring of aircraft systems, ensuring enhanced safety and operational efficiency [19].

Li et al. (2022) conducted a comprehensive survey of Digital Twins, emphasizing their impacts, implementations, and the associated challenges across key industries including aerospace. They particularly shed light on real-time data collection, synchronization, and processing issues that may hamper the smooth adoption of Digital Twins in the aerospace industry. Furthermore, the authors propose a roadmap towards standardized, highly interactive Digital Twins with cognitive capabilities, expanding the technological and business horizons of aerospace applications [20].

Similarly, Fei Tao et al. (2019) have reviewed the state-of-the-art of the Digital Twins research. The authors went through the key components, and the development and major Digital Twins applications in industry. Crucially, they identify several challenges, such as data security and interoperability issues, and posit potential directions for future research and development [21].

N. Sarantinoudis (2023) and T. Y. Melesse (2021) discuss the usability of Digital Twins in industrial environments, showing the importance of this research field to bring more adaptability in real-time for manufacturing processes [22,23].

### 2.2. PSO Explanation

PSO works as an algorithm to search for global minimums or maximums, consisting of several particles (swarm size), that move through the space of possible values for a maximum number of iterations.

This search, in turn, follows two main equations, one that governs the displacement velocity and another that governs the displacement of the particles in the search environment. The velocity update is given by Equation (1):

(1) $$V_{i}\left(k+1\right)=\omega V_{i}\left(k\right)+c_{1}r_{1}\left(P_{i}\left(k\right)-X_{i}\left(k\right)\right)+c_{2}r_{2}\left(G_{i}\left(k\right)-X_{i}\left(k\right)\right).$$

While the position update is performed by Equation (2):

(2) $$X_{i}\left(k+1\right)=X_{i}\left(k\right)+V_{i}\left(k+1\right),$$

where:

- $V_{i}\left(k\right)$: particle velocity *i* at time *k*.
- $X_{i}\left(k\right)$: particle position *i* at time *k*.
- $P_{i}\left(k\right)$: best individual particle position *i* at time *k*.
- $G_{i}\left(k\right)$: best global swarm position at time *k*.

### 2.3. Parameters Estimation Using PSO

In the world of computational modeling, accurate parameter estimation is pivotal for predicting system behavior and ensuring optimal performance. Particle swarm optimization (PSO) has become a popular method for parameter estimation largely because of its conceptual simplicity and computational efficiency. Instead of using complex mathematical gradient computations as in traditional optimization methods, PSO uses a population of candidate solutions (particles) that adjust their positions in the solution space based on their individual experiences and those of their neighbors. This heuristic approach allows for quick convergence to a solution while reducing the risk of getting trapped in local optima [15].

He et al. (2006) used the PSO for estimating chaotic systems parameters. This study shows the capacity of PSO to solve multi-dimensional optimization problems [24].

A study by Schwaab et al. (2008) employed PSO for the estimation of both linear and nonlinear parameters within dynamic systems. An additional advantage found was that the PSO approach could also provide likelihood confidence regions of model parameters very easily [25].

Robotic systems, given their complex interactions, require precise parameter estimation. Zha et al. (2019) utilized a PSO approach to estimate the parameters of a robotic exoskeleton model. The authors also evaluated PSO-hybrids approaches and found that they significantly improved the accuracy of the model predictions compared to other estimation methods [26].

PSO has also been successful in domains like power systems, process control, and robotics. For instance, Polsena et al. (2021) proposed a PSO-based approach for estimating parameters of a DC motor model and achieved accurate real-time parameter estimation, underscoring the adaptability of PSO in diverse settings [27].

Some recent works also discuss the ability of PSO and its variations to accomplish parameter estimation in energy generation applications [28,29] and engineering problems [30].

### 2.4. Discovery of Unknown Components

In the context of industrial systems, even minor variations or the presence of an unidentified component can yield profound implications. Accurately discovering these elusive elements is crucial, as these systems operate under exacting conditions. Minute deviations can cascade into significant outcomes, impacting everything from product quality to system safety. For instance, an uncharted variation in one component on a production line might cause inefficiencies downstream, compromise end-product quality or, in the gravest scenarios, result in equipment malfunctions and safety breaches.

Among the techniques championed in recent years, Brunton et al. (2016) proposed a powerful methodology to discover and characterize the dynamics of nonlinear systems from the available data [31]. However, it typically requires a significant amount of high-quality data to accurately identify the system dynamics, which can be a limiting factor in certain applications.

On the other hand, Deng (2009) used PSO to identify unknown components in a chemical reaction network [32]. The author demonstrated that this approach could properly identify mathematical components of the system based on a series of simple mathematical meta-models.

## 3. Proposed Methodology

**Figure 1:**
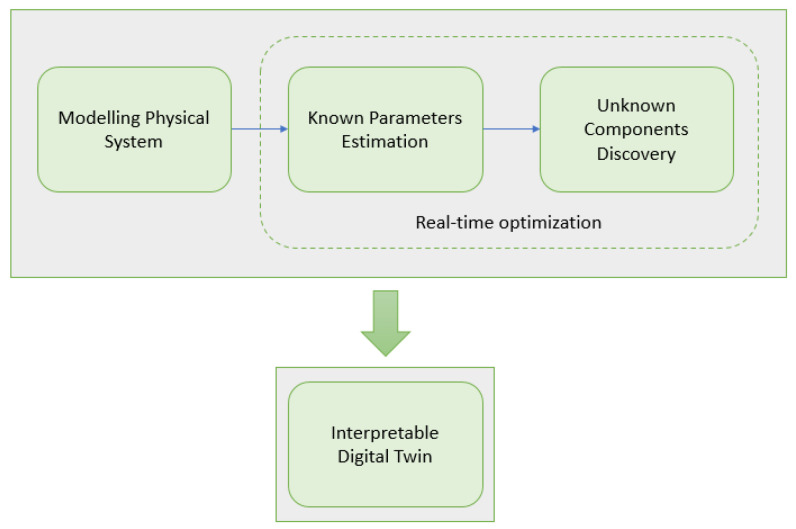
Interpretable Digital Twin creation methodology.

The proposed methodology aims to create an interpretable Digital Twin that integrates online parameter estimation and unknown components discovery using particle swarm optimization (PSO). The main objective is to develop a self-aware machine for adapting its operation so that it evokes better real-time outcomes, by leveraging the benefits of Digital Twins and PSO-based methods. The proposed methodology involves three main steps: (i) modeling the physical system and creating a Digital Twin (DT), (ii) performing online parameter estimation using PSO, and (iii) identifying unknown system components also using PSO. The final end result of the proposed methodology is an interpretable Digital Twin (DT″) that can be used for simulation, optimization, and decision-making purposes. Figure 1 depicts the three steps of the methodology. The blocks inside the dashed line represent the steps of real-time optimization and are the fulcrum of this work.

**Figure 2:**
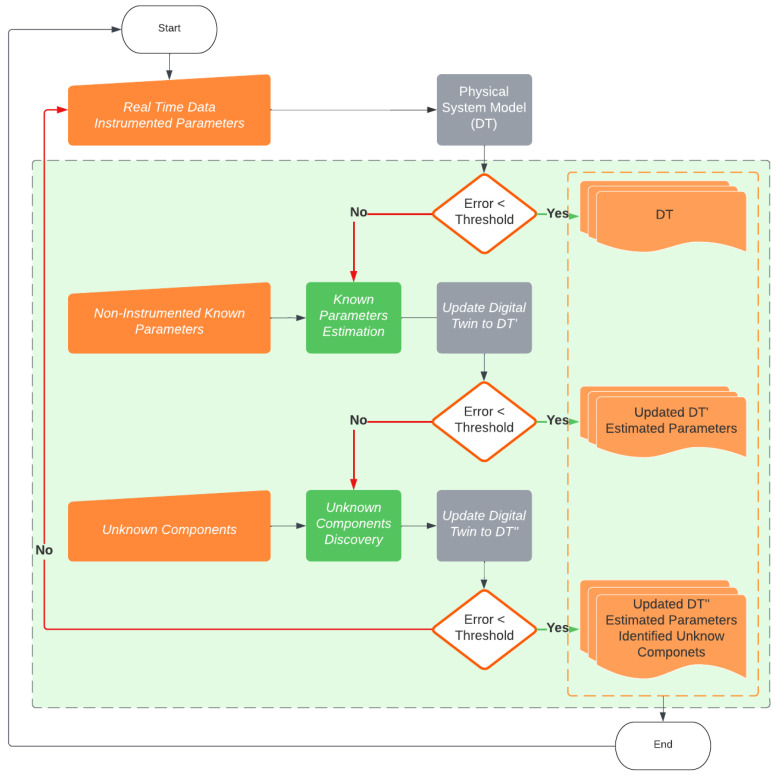
Detailed flowchart of the proposed methodology.

Figure 2 shows the process details. The green dashed area represents the real-time optimization. In summary: first, the real-time data are acquired from the system; then, based on the prediction error, an optimization is performed to estimate the non-instrumented known parameters and update the Digital Twin to DT′. After that, also based on the prediction error, unknown components and parameters are discovered by a second optimization and the Digital Twin is updated to DT″. If the updated model does not satisfy the exit criteria, the process starts again. Finally, a continuous loop is performed with new acquired data. The next sections detail the development steps of our proposed method.

For performing online parameter estimation, both swarm algorithms (as PSO), and genetic algorithms (GA) solve complex optimization problems and could be used interchangeably. However, the choice for PSO was only because we are not currently interested in the evolutionary combinatorial aspect of the solution, which would refer the choice to GA. As the parameters to be optimized are numeric and in a decimal scale, PSO appears to be the most appropriate choice, as it performs searches in continuous functions better than GA.

**Figure 3:**
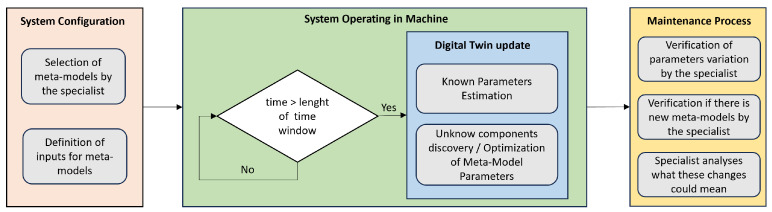
Interpretability process.

The interpretability process offered by the methodology is represented in Figure 3. This interpretability and decision-making process contains one automatic step (system operating in machine) and two steps that require the specialist intervention (system configuration and maintenance process). In this workflow, the specialist needs to add his knowledge related to the system when choosing the meta-models that will be evaluated in the automatic step, adjusting their way of input and output and whether they are additive or multiplicative. In the system operating in machine step, the Digital Twin tries to follow the modifications that are occurring in the real system and inform using the knowledge previously inserted into the configuration by the specialist, about where and how the changes occurred. The last step, in turn, consists of the specialist’s evaluation of the information offered by the intelligent system and the maintenance decision-making.

### 3.1. Digital Twin Modelling (Step-1 of the Proposed Methodology)

Although other techniques can be used, the proposed methodology recommends creating the Digital Twin based on mathematical equations and physical components of the real system. This approach ensures that the Digital Twin model accurately represents the known behavior of the physical system and is easily interpretable.

By using mathematical equations, it is possible to capture the underlying physical principles that govern the system’s behavior and simulate its response to different operating conditions. This approach has been widely used in the development of Digital Twins for various applications, including manufacturing, robotics, and aerospace, among others [33,34].

The model shall be designed to be interpretable, allowing the user to understand how the Digital Twin operates and how it relates to the real system. It shall also be designed to be parameterizable, for updating the system parameters, and modular, allowing for the addition of new components and subsystems as required.

By using this approach, the Digital Twin can accurately capture the underlying physical principles and simulate the system’s response to various operating conditions. Moreover, this approach allows for greater flexibility and adaptability, as the model can be updated with new data or modified to include additional subsystems as needed.

### 3.2. Parameters Estimation and Digital Twin Update (Step-2 of the Proposed Methodology)

The second step of this methodology is to perform the parameter estimation, which consists of using the acquired input and output data as a reference in an optimization algorithm for a given objective function. The algorithm searches for the most suitable model parameters that minimize the error between the real system and the model outputs.

The process outputs of dynamic systems, such as industrial machines, are influenced not only by its inputs but by the process states and variables over time. Hence, the optimization problem needs to take into consideration a time frame within which to calculate the fitness function value based on the model and real system behavior.

The fitness function used in this work is based on the root mean square error (RMSE) between the acquired reference data and the model outputs. The RMSE is a commonly used metric for evaluating the accuracy of a model’s predictions. It is defined as the square root of the average of the squared differences between predicted and actual values.

Hence, in order to evaluate a set of parameters, the model is executed with the parameters generated by the PSO, the input reference values, and the sampling time for the needed iterations. Finally, the RMSE is calculated between the acquired reference data and the model outputs. Hence, the PSO algorithm intends to minimize the RMSE by adjusting the model parameters for the next iteration until a convergence is achieved, which will lead to better predictions and improved system performance.

Once the parameters are estimated, the Digital Twin model is updated with the new optimum values and the system is now able to better predict the machine’s behavior and it is ready for a new acquisition time. Finally, the deviation of the estimated parameters over time provides insights into the machine’s healthy state and operational conditions and could be used for diagnosis purposes, among others.

### 3.3. Unknown Components and Parameters Discovery (Step-3 of the Proposed Methodology)

Even though the third step is identifying and adding unknown components to the fixed structure of the Digital Twin model, specific operational conditions not covered on the first selected ideal structure could influence the system output, causing a discrepancy between the real system and the updated model outputs.

Such a discrepancy could be interpreted as non-normal machine behavior, i.e., faulty conditions due to, for example, an unbalanced shaft, introduced friction, or current leaking among others. The identification of factors is crucial not only for control but also for system diagnoses, machine maintenance, and process optimization in industrial applications.

To enhance the Digital Twin capabilities, the output discrepancy could be addressed by complementing the known model structure and estimated parameters with the discovery of unknown components and parameters.

However, the interpretability of the model still plays a significant role in linking the model structure to parts of the real system as well as its fault conditions and behavior. Hence, the unknown model components are introduced as a combination of known and interpretable meta-models.

The meta-models, as discussed by Deng (2009) [32], are typical mathematical models that influence the system outputs. This set of mathematical models was chosen based on the representation of the dynamic system and the engineering meaning; refer to Table 1.

A second optimization is then performed to select a suitable meta-model combination that complements the adaptive model, decreasing the discrepancies between the Digital Twin model prediction and process data.

The adjustment, or optimization, is carried out in three simplified phases. Each step is explained in detail below:

1. Selection of Meta-Models: In Step-1 of the proposed methodology, the specialist needs to select the meta-models that will be used to complement the Digital Twin model. This selection is made based on the operational characteristics of the real system, such as the presence of friction, misalignment, and wear, among others. Each of these behaviors can be represented by a specific mathematical function, as shown in Table 1.
  The specialist can choose all available meta-models and the optimizer will find the configuration parameters that best describe the system, rendering useless those meta-models that are meaningless for the Digital Twin. However, this approach can cause unnecessary computational costs in Digital Twin update cycles.
2. Definition of inputs for meta-models: Inputs for meta-models are defined. These may include data acquisition time, as well as the system inputs, outputs and states themselves. This versatility allows you to take into account several different possibilities.
3. Optimization of meta-model parameters: In the third phase, the parameters of the selected meta-models are optimized so that they represent the process data in the best possible way. This is done by adjusting the parameters of the meta-models to minimize the difference between the Digital Twin model predictions and the process data.

For such optimization, a new objective function is created. The modularity of the Digital Twin proposed in Step 1 comes into play, allowing a modification of the model structure. The Digital Twin is restructured to consist of the previous ideal model combined with the set of selected meta-models.

Each meta-model is linked to the structure through a main coefficient. These coefficients serve as weights, indicating how important each meta-model is for representing the system’s behavior. Finally, these meta-models and their coefficients are estimated using PSO.

The final Digital Twin model will take the form shown in Equation (3):

(3) $$DT^{\prime \prime }=DT\left(p_{1},p_{2},\ldots ,p_{n},x_{1},x_{2},\ldots ,x_{m}\right)+\sum U_{i}\left(z_{i1},z_{i2},\cdots ,z_{ik}\right),$$

where DT is the Digital Twin’s previously selected model with *n* estimated parameters and *m* inputs, and $U_{i}$ represents each mathematical meta-model with their own *k* number of parameters.

### 3.4. Estimation Parameter Convergence

In each iteration of the search, the PSO particles move in the search space, suggesting new parameters that are tested in the Digital Twin. In the proposed method, PSO seeks to find the set of parameters of the physical model equations that minimize the error (RMSE) between the output values measured during the previous time window and those calculated by the Digital Twin updated with the parameters proposed by the PSO particles in each iteration. As the iterations go by, and following the two equations that govern the PSO, the tendency is for a new value to be found for the Digital Twin parameters that minimize the RMSE.

## 4. Case Study

To validate the methodology, two common industrial machines were selected: (1) DC motor and (2) hydraulic actuator. In addition to their high usability in industry, these two machines are traditional research objects and commonly used to validate control, analysis, and fault detection methodologies [35,36,37,38,39,40,41,42,43].

### 4.1. Industrial DC Motor

The DC motor is one of the most common industrial machines; it is widely used in various applications such as propulsion systems, elevators, and robots due to its simplicity, reliability, and ease of control. However, DC motors are subject to various types of faults that cause deviations in the motor’s behavior and affect its performance. This work will focus on developing a Digital Twin model for a DC motor as a case study.

The electrical equations for the DC motor can be represented as shown in Equation (4):

(4) $$V_{a}=R_{a}I_{a}+L_{a}\frac{dI_{a}}{dt}+K_{e}\omega_{r},$$

where $V_{a}$ is the armature voltage, $R_{a}$ is the armature resistance, $L_{a}$ is the armature inductance, $I_{a}$ is the armature current, $K_{e}$ is the electromotive force constant, and $\omega_{r}$ is the rotor speed.

The mechanical equations can be represented in Equation (5):

(5) $$J\frac{d\omega_{r}}{dt}+D\omega_{r}=T_{load}-K_{t}I_{a},$$

where *J* is the moment of inertia, *D* is the viscous damping coefficient, θ is the angle of rotation, $T_{load}$ is the load torque, and $K_{t}$ is the torque constant of the motor.

Variations in these parameters may indicate failure conditions. For example, a significant change in armor resistance $R_{a}$ may indicate a short circuit, while a variation in the electromotive force constant $K_{e}$ may suggest faults in the commutator or magnetic field windings [44].

**Figure 4:**
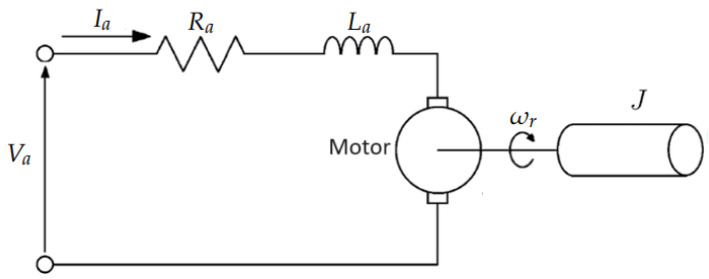
DC motor model diagram.

A diagram to better illustrate the DC motor model is shown in Figure 4.

### 4.2. Hydraulic Actuator

Hydraulic actuators are essential in several sectors, such as aerospace, automotive, and heavy machinery, due to their ability to generate high forces and precisely control the position of the actuators. However, they are exposed to a variety of failures and adverse operating conditions, such as temperature variations, seal wear, and leaks, which can significantly impact their performance [45].

The hydraulic system proposed as a study case is composed of a constant displacement pump connected to the inlet of a piston cylinder and a control valve that, in turn, connects to the fluid reservoir. The position of the piston is then measured by a sensor that informs it to the valve controller.

In this system, the constant displacement pump produces a fluid flow $q_{pump}$ towards the cylinder, while the cross-section of the valve orifice $A_{valve}$ is adjusted to control the flow $q_{valve}$. This control directly influences the speed and displacement of the piston inside the cylinder and, consequently, the displacement of the load.

Assuming that $p_{system}$ is the system pressure in pressure units, the velocity of the fluid in the valve $v_{valve}$ can be estimated by Bernoulli’s law, according to Equation (6):

(6) $$v_{valve}=\sqrt{2\cdot p_{system}},$$

which describes the relationship between system pressure and fluid velocity in the valve. This indicates that the fluid velocity is proportional to the square root of the system pressure.

This is a simplification of the Bernoulli equation, which is valid in situations where energy losses are negligible, there is no significant variation in height, and flow velocities are not extremely high. In this approximation, potential energy is neglected, and only static pressure is considered as a source of kinetic energy.

Thus, the flow through the valve can be expressed as:

(7) $$q_{valve}=A_{valve}\cdot \sqrt{2\cdot p_{system}}.$$

Using Equation (7), it is possible to estimate the flow through the valve based on the cross-sectional area and system pressure. This relationship is important for the sizing and control of hydraulic systems, allowing the evaluation and adjustment of the fluid flow through the valve according to the system’s needs.

The piston velocity, denoted as *v*, can then be obtained by Equation (8):

(8) $$v=\frac{q_{pump}-q_{valve}}{A_{piston}},$$

where $A_{piston}$ is the area of the piston.

The position of the piston over time can be obtained by integrating the velocity, which is shown in Equation (9):

(9) x(t)=∫vdt.

Equation (10) allows for the calculation of the pressure in the hydraulic system based on the forces involved and the area of the piston. This pressure is an indication of the force exerted by the fluid on the piston, considering the effects of load, elastic force, damping force, and internal resistance force. The pressure in the system can be calculated by adding the forces acting on the piston and dividing by its area.

(10) $$p_{system}=\frac{F_{load}+F_{spring}+F_{amort}+F_{resist}}{A_{piston}},$$

where $F_{load}$ is the load force, $F_{spring}$ is the elastic force, $F_{amort}$ is the damping force, and $F_{resist}$ is the internal resistance force.

These equations, derived from the fundamental laws of fluid dynamics and motion, allow the behavior of the hydraulic actuator to be simulated in different scenarios.

**Figure 5:**
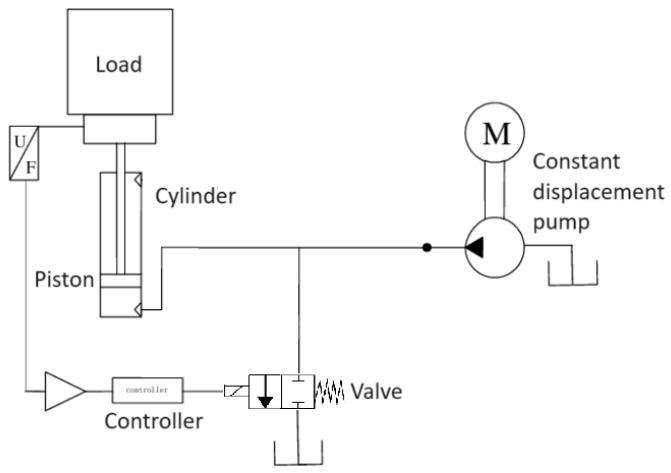
Hydraulic actuator model diagram.

A diagram to better illustrate the hydraulic actuator model is shown in Figure 5.

## 5. Simulation and Results

In this section, we aim to critically assess the capabilities of the Digital Twin, focusing on its adaptability, representation of the real system, and the machine’s self-awareness. Our guiding hypothesis posits that the Digital Twin will not only be able to accurately mirror the real system but will also seamlessly adapt to shifts in operational conditions, environmental changes, and physical alterations during execution using system data.

In order to conduct this evaluation, two case studies were selected: a DC motor (Direct Current) and a hydraulic actuator. Both are components widely found in various industrial contexts and, as highlighted in the literature, present significant challenges in terms of maintenance and control due to the variety of failures and operational conditions they can face [27,46,47,48].

DC motor and hydraulic actuator models are created and simulated in a virtual environment. Through these models, we intend to represent the behavior of real systems, replicating their operations and variability as much as possible. Furthermore, adjustments were made to model parameters and fault insertions to evaluate the Digital Twin’s ability to adapt to different operating conditions such as load changes, increased friction, wear, etc.

In the same virtual environment, the machine controller is implemented according to the proposed methodology. This controller is used to provide the necessary inputs for models of real systems, acquire system input and output data—such as voltage, current, force, temperature and displacement—and simulate the implementation of the Digital Twin. The performance and adaptability of the Digital Twin are compared with the behavior of the simulated real system to assess its accuracy and ability to faithfully represent the system.

**Figure 6:**
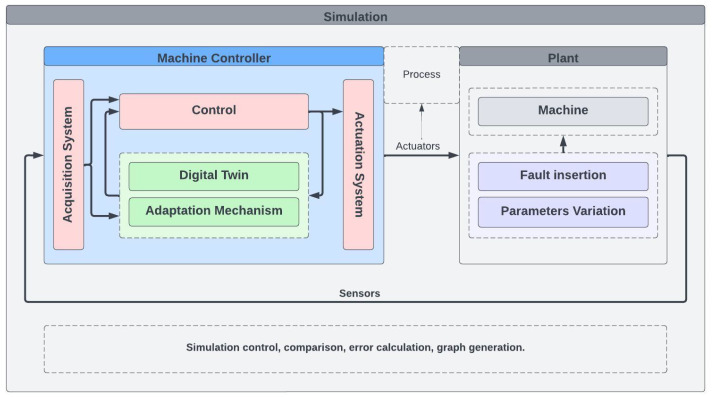
Diagram of the virtual simulation environment.

Figure 6 illustrates a block diagram of the virtual simulation environment. Three different parts of the environment are shown: the machine controller, the plant, and the simulation control.

The machine controller is composed of the Digital Twin and the adaptation mechanism, both already discussed previously. The Digital Twin is responsible for representing the machine in question, while the adaptation mechanism ensures that the model can adjust itself according to changes in operating conditions and parameter variations. The machine controller also houses the control and actuation system, which interacts directly with the Digital Twin, providing the necessary inputs to the machine and collecting the output data. The controller also includes the data acquisition system, which captures information relevant to the operation of the machine.

The plant, in turn, houses the machine simulation. This simulation, which represents the real physical system, is capable of reproducing the behavior of the machine, taking into account its characteristic parameters and the inputs provided by the controller. Furthermore, the plant allows the insertion of faults and parameter variations, simulating different operating conditions and scenarios that the machine may face.

Finally, simulation control coordinates the task execution order, time step control, simulation loop, error calculation, signal storage management, and graph generation. These processes help in analyzing the results and adjusting the simulation according to the research needs.

### 5.1. Simulation Setup

An experimental and simulation setup was developed using a virtual environment and Python programming language. The DC motor model will represent the real machine behavior and it is executed as a time-discrete function running in a time step of 0.0001 s, where the inputs are the armature voltage and the load torque, and the outputs are the armature current and the shaft speed. Changes to the motor’s parameters as well as fault insertions as unbalanced shaft and friction increases will be performed to evaluate the system at different operational conditions.

Moreover, the virtual controller is used to acquire input and output data at every 0.01 s. After the acquisition time is complete, the Digital Twin attempts to adapt itself to the changing conditions. We will compare the Digital Twin’s behavior and adaptability to the real system’s behavior to assess its accuracy and ability to represent the system. This same acquiring time of 0.01 s is used as sampling time to fill a data buffer of 200 registers, i.e., 2 s of simulation. Every 200 registers, the Digital Twin actualization routine goes on. The pymoo library [49] is used for the PSO algorithm; its parameters are depicted in Table 2 for both cases. In both study cases, the optimization constraints were tuned based on their physical limitations parameters, ensuring they remain within realistic bounds.

These parameters were refined by initial experiments, allowing the algorithm to offer a better performance in case studies; in particular, the increase in the parameter of swarm size—which refers to the number of particles in the PSO population—in addition to the selected value did not represent a significant improvement that would justify its use.

### 5.2. DC Motor Model

As discussed before, the DC motor model is based on the mathematical equations that describe its behavior. The differential equations were rearranged as discrete-time functions using the numerical integration method with $i_{k}$ and $\omega_{k}$ as the armature current and rotor speed outputs of the model at instant *k*, respectively. The differential equations of $i_{k}$ and $\omega_{k}$ are (11) and (12), respectively. The inputs are the armature voltage ($V_{a}$) and the load torque ($T_{load}$), the previous values of current ($i_{k-1}$) and speed ($\omega_{k-1}$), and the integration time step.

The physical DC motor parameters are defined as follows:

(11) $$i_{k}=i_{k-1}+T_{s}\cdot \frac{V_{a}-R_{a}\cdot i_{k-1}-K_{e}\cdot \omega_{k-1}}{L_{a}}$$

(12) $$\omega_{k}=\omega_{k-1}+T_{s}\cdot \frac{i_{k}\cdot K_{t}-T_{load}-D\cdot \omega_{k-1}}{J}.$$

$R_{a}$: Armature resistance in Ω.

$L_{a}$: Armature inductance in H.

*J*: Rotor moment of inertia in $\mathrm{kg}\cdot m^{2}$.

$K_{e}$: Electromotive force constant in V·s/rad.

$K_{t}$: Motor torque constant in Nm/A.

*D*: Rotor damping coefficient in Nm·s/rad.

For the analysis, a small-to-medium-sized DC motor was selected, with parameters that are commonly observed in motors of similar sizes and types, thus increasing the applicability of the results to a wide range of motors in diverse real-world applications.

The DC motor’s initial parameters are depicted in Table 3.

The speed set-point is a step function that changes between 0 rad/s, 5 rad/s, 10 rad/s, 15 rad/s, and 20 rad/s at different times. The speed is controlled using a proportional-integrative-derivative (PID) controller with gains $K_{p}=0.3$ and $K_{i}=0.1$ that adjusts the motor voltage input. The load torque is proportional to the rotor speed with a coefficient of 0.006Nm·s/rad. The estimation time is evaluated as 2 s, 4 s, and 10 s so the suitable time frame could be selected.

Three scenarios were designed to simulate various types of operational conditions, environmental factors, physical changes, or degradation that can occur in a DC motor system. The first two, i.e., resistance armature degradation and bearing wear, evaluate the parameters estimation step. On the other hand, the unbalanced shaft evaluates the unknown components discovering capabilities.

In the resistance armature degradation scenario, some deviation is introduced in the armature resistance to simulate the degradation of this component. A 1200-s simulation takes place while $R_{a}$ is decreased over time from 6.5 Ω to about 5.6 Ω using an exponential decay −0.044·exp(0.003·t)+0.044, with each time step initiating at 160 s.

In the bearing wear scenario, the damping coefficient (*D*) is degraded to simulate bearing wear. A 1200-s simulation is used while *D* increases over time from the initial value to about 0.00227H, using a an exponential slope 0.00003806·exp(0.003·t)−0.00003806 with each time step initiating at 160 s.

To simulate the unbalanced shaft, deviations are introduced to the rotor speed. A sinusoidal error is added to the speed calculation at each time step. The amplitude of the error is set as $5\;{\mathrm{rad}/s}^{2}$, which is multiplied by the simulation sample time. The frequency is chosen to be fixed at 10 rad/s. Thus, the unbalanced shaft simulation is performed by modifying the $w_{k}$ as shown in Equation (13), where *t* is the time at instant *k*.

(13) $$\omega_{k}=\omega_{k-1}+T_{s}\cdot \frac{i_{k}\cdot K_{t}-T_{load}-D\cdot \omega_{k-1}}{J}+T_{s}\cdot 5\cdot \sin\left(10\cdot t\right).$$

#### 5.2.1. Time Window Estimation

For the different scenarios, the variance in the time window estimation indicates that the acquisition time needs to be in accordance with the system variation rate to capture the system’s dynamics accurately. Specifically, an acquisition time of 2 s yielded the lowest percentage error across all degradation scenarios. Different configurations of sampling data (0.1 s, 1 s, 2 s, 4 s and 10 s) were previously tested using the armature resistance degradation problem, showing better results for the smallest one, but no relevant difference between 0.1 s, 1 s, and 2 s.

#### 5.2.2. Scenario-1: Armature Resistance Degradation

**Figure 7:**
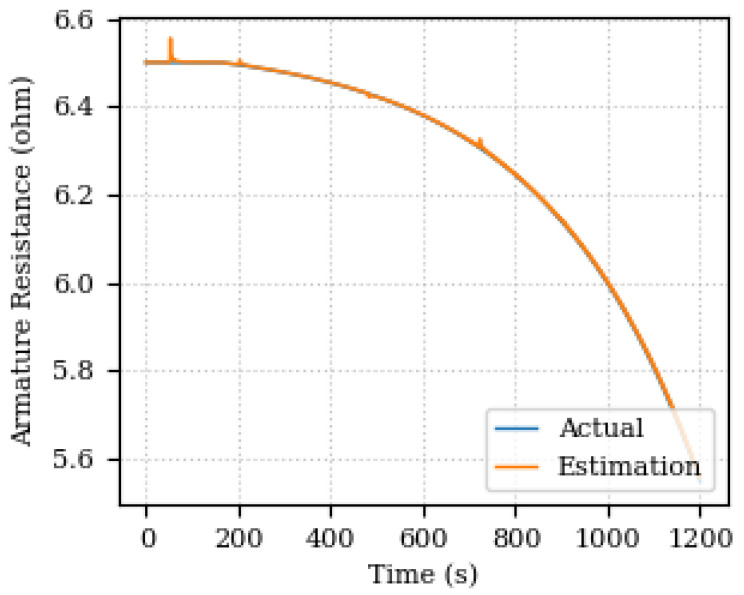
Estimated and actual values of the armature resistance.

**Figure 8:**
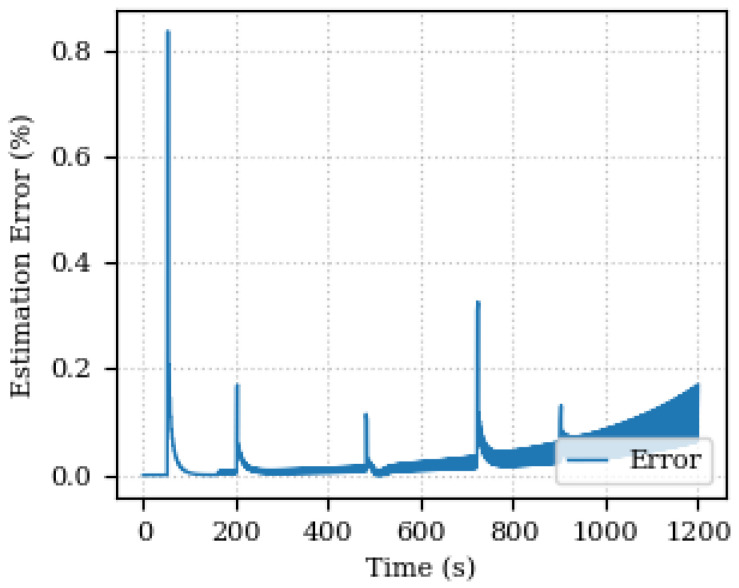
Estimated percentage error for the armature resistance.

The estimation error is shown below 0.2% with peaks at each speed step variation. Figure 7 and Figure 8 show the estimated and actual values of the armature resistance and the estimation percentage error, respectively.

**Figure 9:**
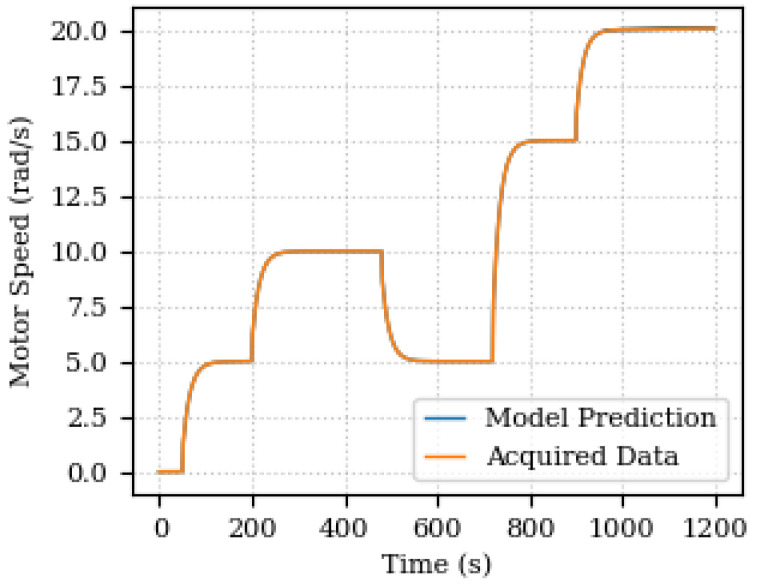
Predicted and acquired data for output speed.

The predicted and acquired data for output speed are depicted in Figure 9. The results show a good agreement between the predicted and acquired data, indicating the effectiveness of the proposed parameter estimation method.

It is possible to realize that the error increases towards the end of the simulation (1200 s). As the acquisition window for updating the Digital Twin is fixed (200 samples), and in the final part of the graphs there is a greater variation in the parameter values, the estimation is compromised from being accurate, since the data indicate that the parameters are changing. The point is that a prediction is not being made of how the system will react, nor even an estimate of trends, but rather a comparison with the values already measured. Therefore, there is a greater discrepancy between the final 200 samples and what was calculated after the parameters were optimized. Therefore the error is greater, due to the variation in the actual measured values also being greater. One way to improve this behavior and reduce the error with increasing variation is to make the acquisition interval follow the variation, also making it adaptable. In this case, decrease the acquisition interval to have a smaller error.

#### 5.2.3. Scenario-2: Bearing Wear

**Figure 10:**
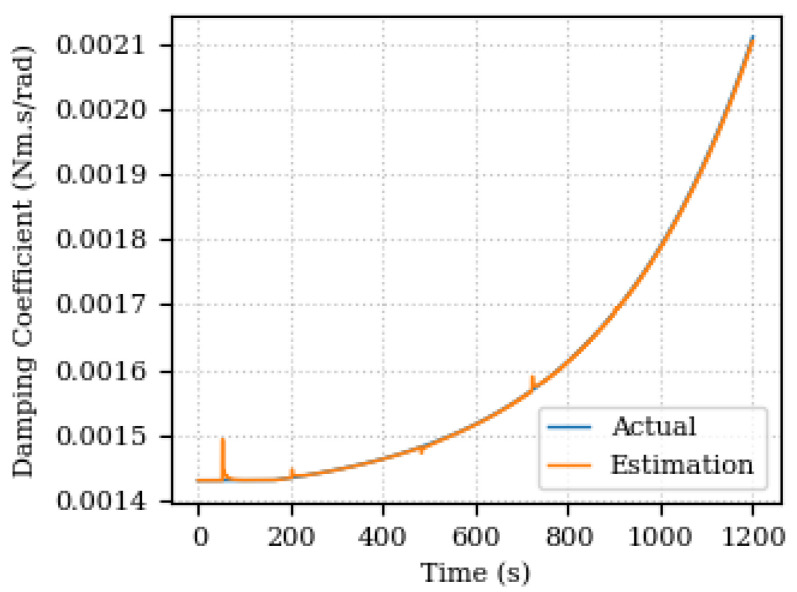
Estimated and actual values of the damping coefficient.

**Figure 11:**
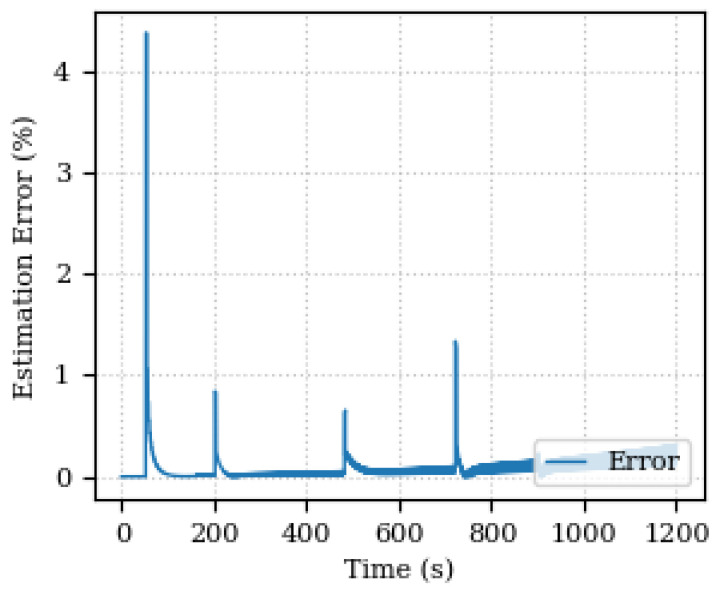
Estimated percentage error of the damping coefficient.

Figure 10 and Figure 11 depict the estimated and actual values of the damping coefficient and its estimation percentage error, respectively. In this case, the error is found below 0.5% with some peaks again on the speed step changing.

#### 5.2.4. Scenario-3: Unbalanced Shaft

**Figure 12:**
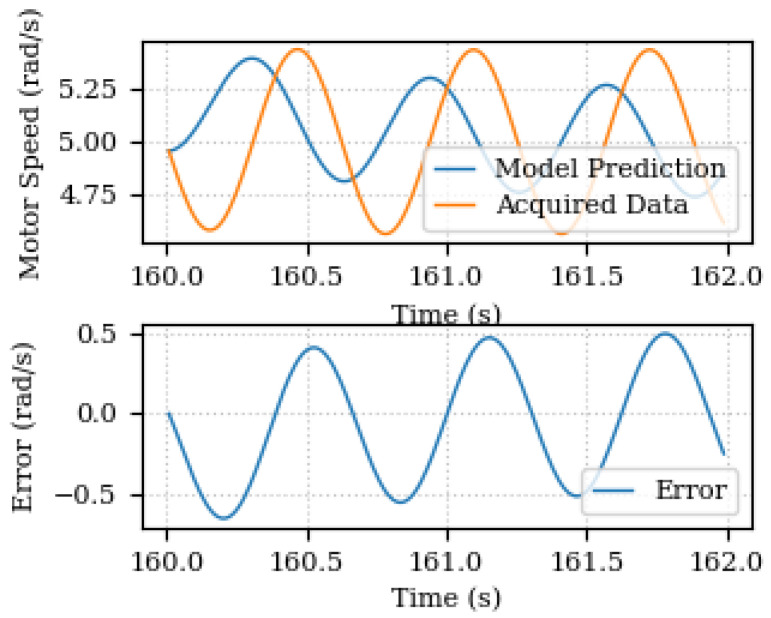
Predicted and acquired data for output speed before unknown components discovery.

**Figure 13:**
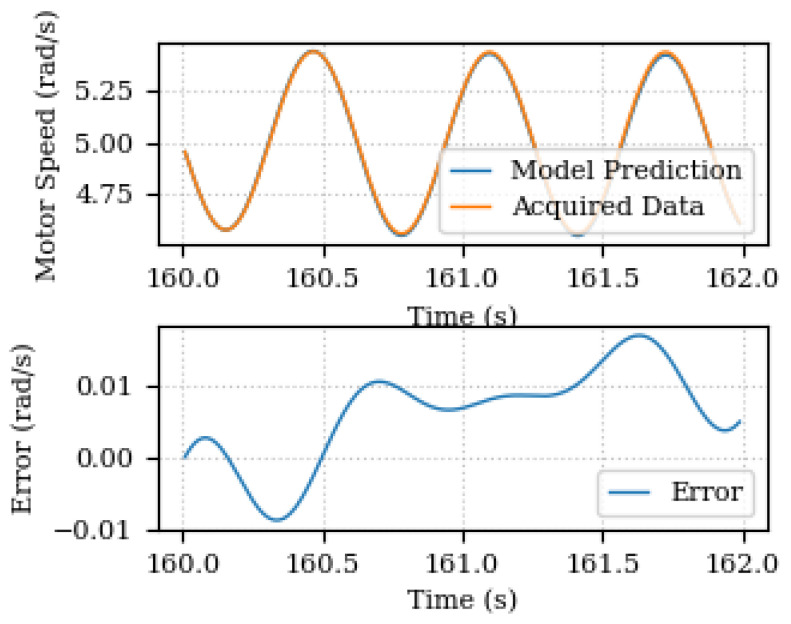
Predicted and acquired data for output speed after unknown components discovery.

When introducing the speed sinusoidal error, the parameter estimation is not enough to guarantee the model prediction performance as shown in Figure 12. Once the unknown components discovery takes place, the updated model is capable of better predicting and representing the plant behavior. Figure 13 depicts the model prediction and the prediction error of the new updated model for a 2 s estimation time window. It shows that the maximum absolute error is reduced from 0.5 rad/s to less than 0.02 rad/s.

The unknown components discovery has deactivated the less significant meta-models and added the periodic function to the previous model structure. The discovered complementary model for that estimation time window was 4.91985774·sin(−9.97780433·t−0.46427915) and the calculated cost was equal to 0.01620499.

### 5.3. Hydraulic Actuator Model

Analogous to the previous example, this case addresses the modeling of a simplified hydraulic system, which includes a constant displacement pump, a controlled valve, and a piston cylinder. Euler’s method was used to transform the physical continuous differential equations to a discrete-time model, as in Equation (9).

(14) $$x_{k}=x_{k-1}+T_{s}\cdot v_{k-1},$$

where $T_{s}$ is the integration time step and *k* is the index that denotes the discrete time instant. The position of the piston at instant *k* is given by its position at the previous instant plus the product of the piston velocity at the previous instant and the time step.

Substituting the velocity Equation (8) as a function of discrete time, we have Equation (14),

(15) $$x_{k}=x_{k-1}+T_{s}\cdot \frac{q_{pump}-A_{valve}\sqrt{2\cdot p_{k-1}}}{A_{piston}}.$$

The model’s outputs are the system pressure $p_{k}$ and the piston position $x_{k}$ at each instant *k*, and the inputs are the controlled cross-section of the valve $A_{valve}$, the load force $F_{load}$, the position $x_{k-1}$, and the pressure $p_{k-1}$ of the previous instant, and the time step of integration Ts.

Thus, the pressure at instant *k* is given by Equation (16):

(16) $$p_{k}=\frac{F_{load}+F_{spring}+F_{amort}+F_{resist}}{A_{piston}},$$

where $F_{spring}=k_{spring}\cdot x_{k}$ and $F_{amort}=D\cdot v_{k}$ and represent the spring and shock absorber forces, respectively.

The physical parameters of the hydraulic system are defined as follows:

$k_{spring}$: Elastic constant of the system in N/m.

*D*: System damping coefficient in N/(m/s).

$q_{pump}$: Constant displacement pump flow in $m^{3}/s$.

$A_{max}$: Maximum cross-section of the Valve in $m^{2}$.

$A_{piston}$: Piston area in $m^{2}$.

The initial parameters of the selected hydraulic actuator, along with their corresponding units, are presented in Table 4. These parameters were determined based on typical considerations of an automotive elevator system and were established to provide a good balance between model simplicity and reasonable representation of hydraulic behavior.

In order to simulate the rise and fall cycles of the actuator, such as a hydraulic elevator, the position reference value is a sinusoidal function with amplitude 0.45 m, and frequency 0.005 Hz.

To simulate system input, the load force was set as a fixed vertical load of 1000 kg. The estimation time is evaluated with three different values, 2 s, 4 s and 10 s so that the appropriate time interval can be determined.

The actuator position is controlled by changing the cross-sectional area of the valve using a PID controller with the gains $K_{p}=0.000346$, $K_{i}=3.513406$, $K_{d}=0.000119$ and filter factor N=2.076743 to control the cross-sectional area of the valve.

Equation (17) represents the calculation of the controlled cross-section of the valve *A*, based on the PID controller in its discrete form.

(17) $$A=Error\cdot \left(k_{p}+k_{i}\cdot T_{s}\cdot \frac{1}{z-1}+k_{d}\cdot \frac{N}{1+N\cdot T_{s}\cdot \frac{1}{z-1}}\right).$$

The equation incorporates proportional, integrative, and derivative terms for control, represented by the coefficients $k_{p}$, $k_{i}$ and $k_{d}$, respectively. $T_{s}$ is the integration time step. The term *z* represents the forward operator in the Z domain, used to model the discrete dynamics of the system. The term z−1 denotes a one-sample delay, common in discrete systems.

This control adjusts the cross-section of the valve *A* by the Error, between the desired and actual position. The values of the PID controller parameters were determined through simulation and tuning techniques, aiming to obtain optimal performance of the control system.

**Figure 14:**
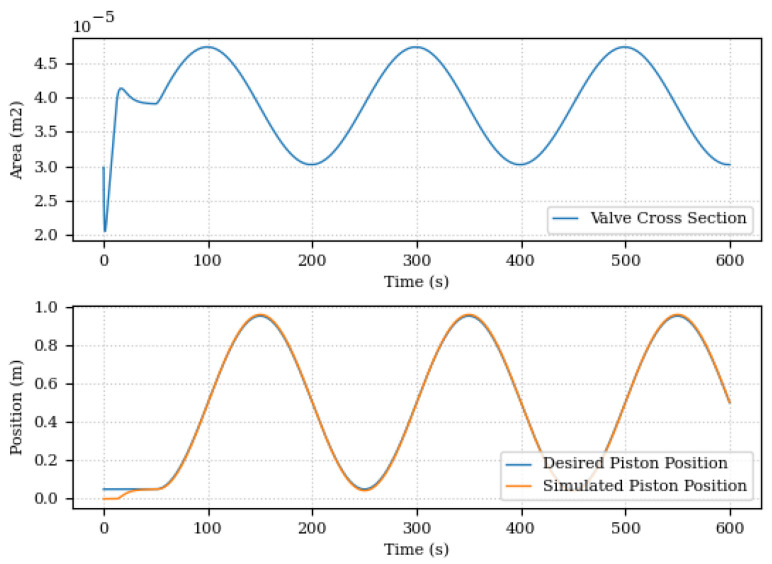
Hydraulic actuator position control.

Figure 14 illustrates the system position control input, that is, the cross section of the valve, and the desired and simulated position of the actuator. This desired position profile is used throughout the next assessments.

Two scenarios were devised to simulate different operating conditions or physical changes to components that may occur in this type of system. These scenarios provide an important context for testing and analyzing the robustness and accuracy of the Digital Twin.

The first scenario proposes a change in the viscosity of the hydraulic fluid, which directly reflects on the system’s damping coefficient. This condition may be representative of a situation in which the degradation of the hydraulic fluid occurs, causing an increase in viscosity and, consequently, in the damping coefficient. Factors such as aging, contamination, and variations in temperature and pressure can cause this degradation [50].

To simulate this condition, the damping coefficient *D* was allowed to gradually increase over a 600-s simulation. From the initial value to about 100N/(m/s) using an exponential slope 0.044·exp(0.003·t)−0.044 at each time step starting at 160 s, initial moment of degradation.

Then, the second scenario considers the occurrence of a fluid leak in the system. The objective of this scenario is to test the “discovery of unknown components” capability of the Digital Twin, that is, to verify whether the model is capable of accurately identifying and modeling the occurrence and impact of the leak on system performance.

To simulate this condition, the original model was modified to include a leakage term. This component, which is initially unknown to the Digital Twin, is added to the original model of the hydraulic actuator plant, specifically to the system pressure update equation, now considering a leakage fraction. Equation (18) shows this new calculation:

(18) $$p_{k}=\left(1+C\right)\cdot \frac{F_{load}+F_{spring}+F_{amort}+F_{resist}}{A_{piston}},$$

where *C* represents the leakage coefficient impacting the system pressure.

The leakage coefficient was set as a fixed value in the model of 0.2, influencing the valve flow rate and overall system pressure.

#### 5.3.1. Scenario-1: Hydraulic Fluid Degradation

**Figure 15:**
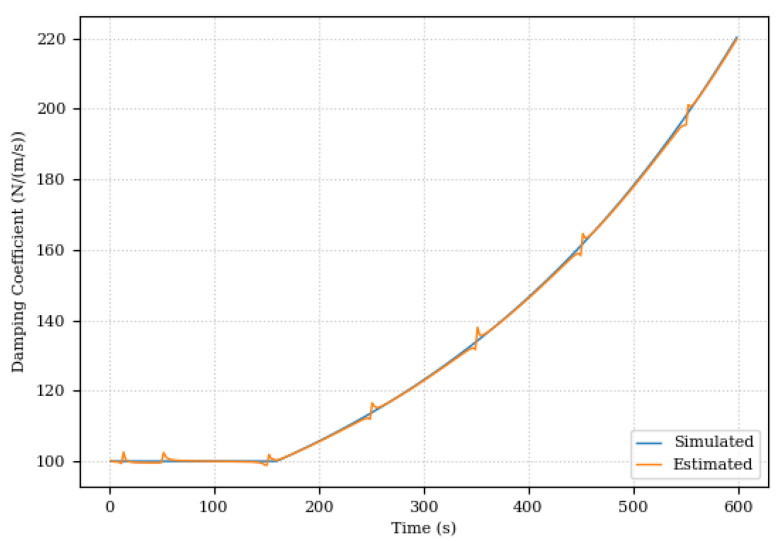
Estimated and actual damping coefficient.

**Figure 16:**
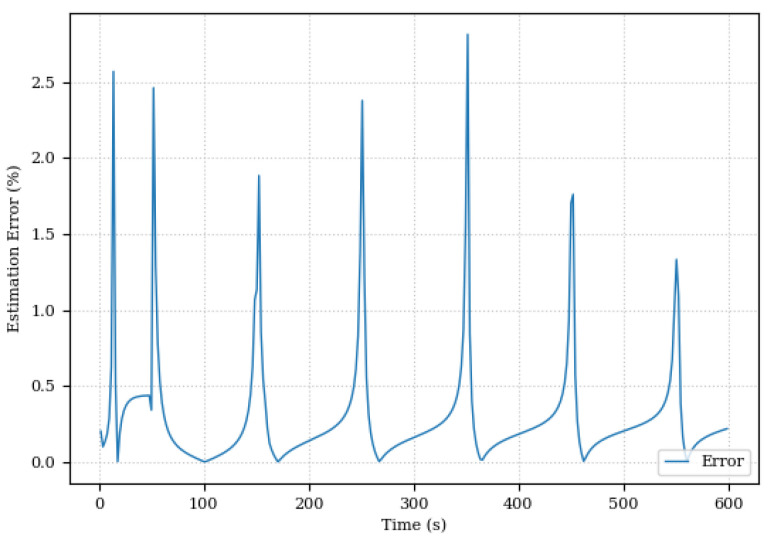
Relative damping coefficient estimation error.

The change in damping coefficient and the estimation error of the relative damping coefficient is shown in Figure 15 and Figure 16.

The relative parameter estimation error was below 3% as shown in Figure 16. It is important to note that the spikes in error can be attributed to the sinusoidal behavior of the system, with a greater error observed when the variation in the acquired data is smaller. These peaks occur precisely when the actuator reaches the maximum amplitude of movement, evidenced by a gradient in the function close to zero, a challenging point in this system.

**Figure 17:**
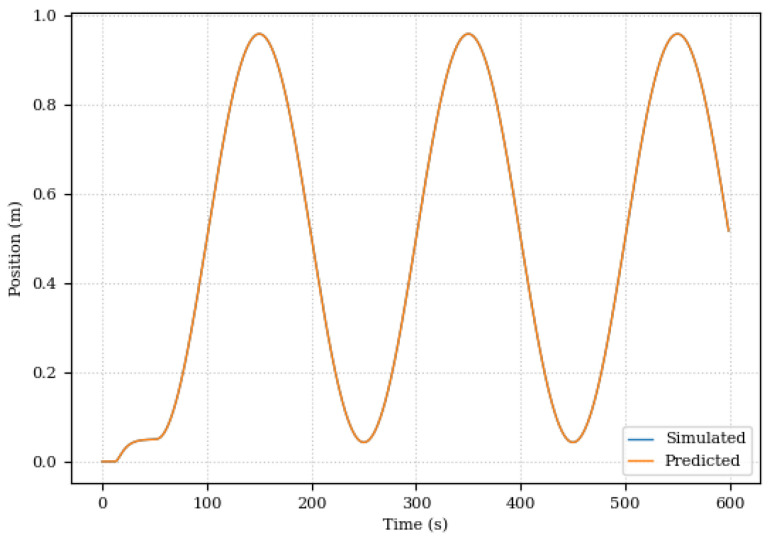
Piston positions, simulated and predicted, for the scenario of changing the damping coefficient.

**Figure 18:**
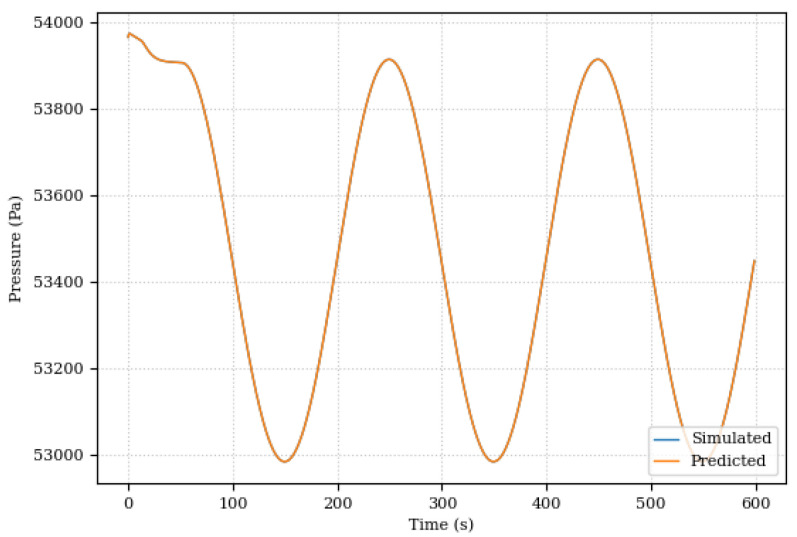
System pressures, simulated and predicted, for the damping coefficient modification scenario.

This first scenario was mainly used to evaluate the Digital Twin’s ability to adapt to changes in the system. In this sense, the results were promising. Even with the changes in the damping coefficient, the Digital Twin was able to adapt and continue to provide accurate predictions for system position and pressure as illustrated in Figure 17 and Figure 18.

The deviation in the damping coefficient leads to the interpretation of a degradation of the hydraulic fluid, a condition that can have significant implications for the operation of hydraulic systems. This reinforces the applicability of the Digital Twin in the health management of machines in industrial environments.

#### 5.3.2. Scenario-2: Internal Hydraulic Fluid Leak

**Figure 19:**
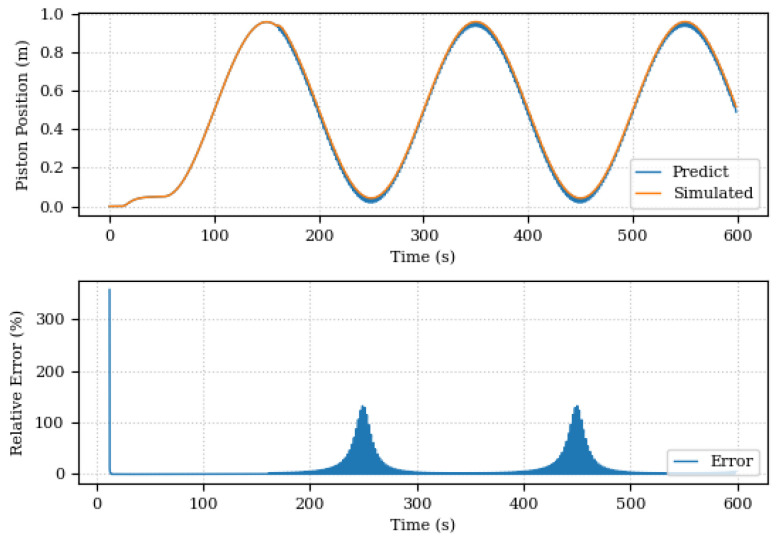
Piston positions, predicted and simulated, before the discovery of unknown components.

**Figure 20:**
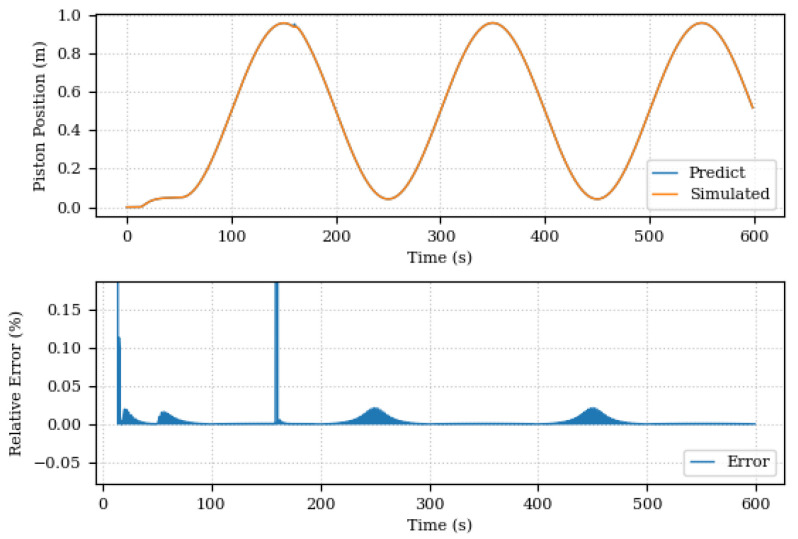
Piston positions, predicted and simulated, after the discovery of unknown components.

Figure 19 and Figure 20 present, respectively, comparisons between the values and errors of pressure and piston position predicted by the Digital Twin and simulated by the system before the discovery of components unknown. The graphs show a large variation in the system pressure predictions by the Digital Twin, from the moment 160 s where system degradation began, although it is possible to observe a general pressure trend in the system, the estimation of model parameters only does not is sufficient to eliminate existing discrepancies in the data. These differences highlight the need to complement the existing model. In the position graph, the error is mitigated by the PID controller.

**Figure 21:**
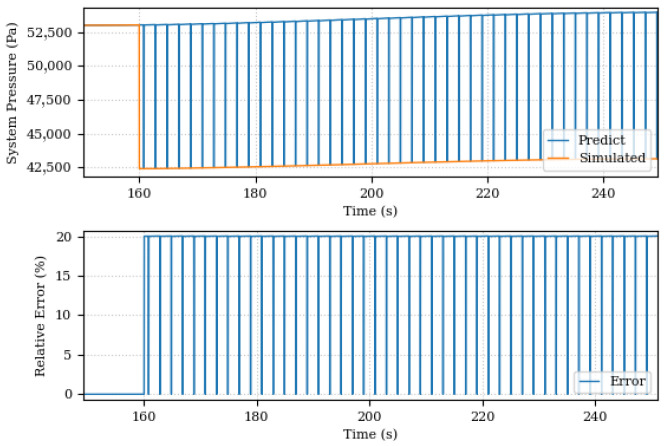
System pressures, predicted and simulated, before the discovery of unknown components.

**Figure 22:**
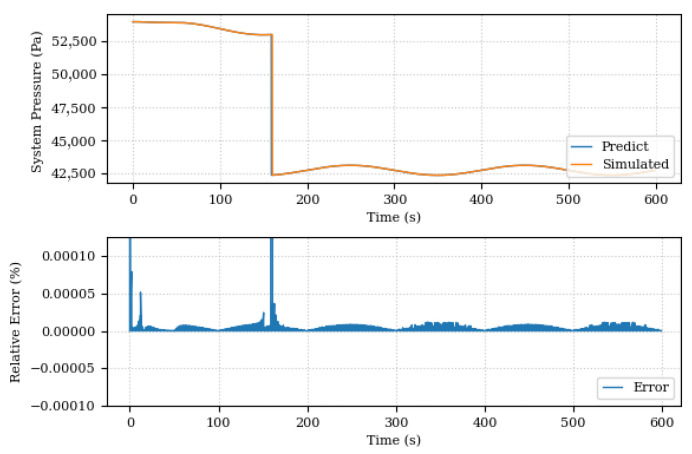
System pressures, predicted and simulated, after the discovery of unknown components.

Figure 21, on the other hand, shows the pressure comparison after the Digital Twin runs the discovery of unknown components routine. The predicted and simulated pressure curves are now superimposed and the relative error between them is small. The same behavior is seen in Figure 22, where the comparison of the system position after the discovery of unknown components is shown. The improvement in forecast accuracy is notable, with the Digital Twin now being able to more accurately monitor changes in the pressure and system position.

The complementary model discovered for this estimation time window is shown in Equation (19), where *C* is the slope of the discovered linear function, and the system pressure becomes the input to this function. In terms of interpretability, the discovered component is in the form of an internal leak in the hydraulic system. Thus, this new term represents the flow rate due to leakage in the system, which is a function of the current pressure Pressure.

(19) C·Pressure.

With the addition of this component, the Digital Twin adjusted to the new system conditions, demonstrating its capacity for self-awareness and adaptability. The new model discovered by the Digital Twin adaptation updates the system pressure through Equation (20), with $p_{k}^{\prime }$ indicating the fixed model output and $p_{k}$ indicating the new system outlet pressure for the adapted model.

(20) $$p_{k}=p_{k}^{\prime }-0.19999991\cdot p_{k}^{\prime }.$$

### 5.4. Summary

The results underscore the Digital Twin’s ability to provide a deep understanding of the operational condition of the DC motor and the hydraulic actuator. By tracking parameters such as armature resistance, bearing wear (DC motor) and hydraulic fluid degradation (hydraulic actuator), the Digital Twin delivers a quantitative and dynamic measure of machine health in real-time.

What sets this Digital Twin apart is its pronounced interpretability. When irregularities such as shaft imbalances (DC Motor) or internal fluid leak (hydraulic actuator) were introduced, the Digital Twin’s model incorporated periodic functions to unveil previously hidden facets of the system. This transparent representation empowers engineers, technicians, and decision-makers to discern the occurrence of issues and their root causes, facilitating informed maintenance decisions.

The discovery of the unknown component also illustrates the interpretability provided by the Digital Twin. That is, it is not just that the Digital Twin has identified a change, but rather that the final adaptive model it provides is such that operators (whether human or machine) can interpret that change in terms of the components and operations of the system. In this sense, the Digital Twin not only signaled that the control effort increased, but provided a model in which the reason for this change could be correctly identified.

In essence, the Digital Twin’s combination of precision and, more crucially, its unparalleled interpretability, establishes it as a pivotal tool for the safe and efficient operation of complex systems, allowing for early fault detection, wear prediction, and proactive maintenance.

## 6. Conclusions

This work contributes to the emerging field of self-aware machines, which, through real-time learning and adaptation, promise to significantly improve the performance of industrial machines. In particular, the main contribution is the proposal and development of a robust methodology for the creation and implementation of interpretable and adaptable Digital Twins. The combination of Digital Twins and methods based on PSO algorithms demonstrates great potential for improving the performance and efficiency of different industrial applications, by means of explainable insights.

Our method’s innovation lies in its integration of physical principles, mathematical formulations, and optimization techniques, ensuring both precise estimations and clear interpretability. The proposed methodology proved to be effective for developing interpretable and adaptable Digital Twins in controlled scenarios. With this methodology, Digital Twins demonstrated their ability to dynamically follow and adjust to changes in the corresponding physical system, demonstrating an adaptability of great importance for a variety of industrial applications. The effectiveness of the methodology was validated in a simulation environment, through two case studies—an industrial DC motor and a hydraulic actuator—and was shown to be capable of operating effectively under a wide variety of conditions. It is important to highlight that, although the case studies are specific, the methodology has the potential to be applied to a wider range of industrial systems and scenarios, thus meeting the objectives established at the beginning of this work.

Another notable point of this work is the emphasis on the interpretability of Digital Twins. Interpretability is a vital feature that ensures that Digital Twins can be used effectively by engineers, technicians, and machine operators. The Digital Twins developed in this work not only adapt and learn in real-time, but also provide valuable and understandable insights into the state and behavior of the physical system.

Within the scope of predictive maintenance, interpretable Digital Twins offer the unprecedented capability to monitor machine health in real-time and predict potential failures, proactively addressing potential issues before they cause significant disruptions. This reduces downtime, maximizing operational efficiency. And, thanks to their ability to adapt in real-time, Digital Twins also play an important role in autonomous operations. They can be used to automate decision-making processes in industrial applications, offering real-time solutions to emerging issues and improving operational efficiency and safety.

Digital Twins can serve as a key component in this context by providing a virtual representation of the physical system that can be used for simulation and optimization. PSO methods have been successfully used for online parameter estimation and component identification, which can optimize the performance of the machine in real-time. While the case study presented in this paper demonstrates the feasibility of using these technologies to develop self-aware machines that can adapt and optimize their performance during its operation, improving the efficiency and reliability of the system, future work will delve into more intricate industrial cases to demonstrate the scalability and adaptability of our proposed methodology.

A possible improvement in the proposed methodology is to make the acquisition interval for optimizing the Digital Twin adaptive to follow the variation in measured values, that is, the greater the variation in measured values, the shorter the acquisition interval. Such improvement can reduce the error between measured and calculated values after optimizing parameters when there is a large variation in measured values.

Furthermore, the final stage of the proposed interpretability process (maintenance process) is not yet an automated sub-process; therefore, it requires the specialist to decide. However, the authors believe that this sub-process can be automated to some degree in future works using some hybrid approach with semi-symbolic artificial intelligence. This new automation can further contribute to achieving self-awareness in industrial environments.

However, building and validating Digital Twin models require accurate and reliable data, and implementing complex optimization algorithms in real-time poses significant challenges. Furthermore, the development of self-aware machines requires interdisciplinary expertise and collaboration between different areas such as machine learning, control theory, and industry. Addressing these challenges and limitations requires further investigations and simulations. Further studies on using the adaptive Digital Twin proposed here can benefit from some ‘freedom of thinking’ on behalf of the control systems, e.g., the addition of some degree of ‘consciousness’.

## Figures and Tables

**Table 1:** Meta-models examples and working conditions.

Model Function	Model Equation	Working Conditions
Linear	mx+b	Friction, misalignment, backlash
Exponential	$ab^{x}$	Current leaking, deterioration, wear and tear, corrosion
Negative exponential	$ae^{-bx}$	Ageing, degradation, fatigue, crack growth
Power	$ax^{b}$	Hard stops, overloading, mechanical shock
Logarithmic	aln(x)+b	Saturation, memory effects, hysteresis
Periodic	asin(bx+c)+d	Unbalanced shaft, shaft misalignment, machine unsteadiness

**Table 2:** PSO optimization parameters.

Parameter	Symbol	Value
Swarm size	$N_{\mathrm{swarm}}$	15
Maximum iterations	$N_{\max}$	1000
Inertia weight	*w*	0.9
Cognitive parameter	$c_{1}$	2.0
Social parameter	$c_{2}$	2.0

**Table 3:** Initial parameters for DC motor simulation.

Parameter	Value
$R_{a}$	6.5 Ω
$L_{a}$	0.673 H
*J*	0.001171 kg·m^2^
$K_{e}$	0.038 V·s/rad
$K_{t}$	0.038 Nm/A
*D*	0.00143 Nm·s/rad

**Table 4:** Initial parameters hydraulic actuator simulation.

Parameter	Value
$k_{spring}$	200 N/m
*D*	100 N/(m/s)
$q_{bomb}$	0.02 m^3^/s
$A_{max}$	$10\cdot 10^{-5}$ m^2^
$A_{piston}$	0.196349375 m^2^

## Data Availability

Data is contained within the article.

## References

[1] Bahrin M., Othman F., Azli N., Talib M. (2016). Industry 4.0: A review on industrial automation and robotic. J. Teknol..

[2] Brown R.J. (2018). A Modern Introduction to Dynamical Systems.

[3] Kuhnle A., Kaiser J.P., Theiß F., Stricker N., Lanza G. (2021). Designing an adaptive production control system using reinforcement learning. J. Intell. Manuf..

[4] Whitcomb L.L., Arimoto S., Naniwa T., Ozaki F. (1997). Adaptive model-based hybrid control of geometrically constrained robot arms. IEEE Trans. Robot. Autom..

[5] Fuller A., Fan Z., Day C., Barlow C. (2020). Digital Twin: Enabling Technologies, Challenges and Open Research. IEEE Access.

[6] Jafari M., Kavousi-Fard A., Chen T., Karimi M. (2023). A Review on Digital Twin Technology in Smart Grid, Transportation System and Smart City: Challenges and Future. IEEE Access.

[7] van Dinter R., Tekinerdogan B., Catal C. (2022). Predictive maintenance using Digital Twins: A systematic literature review. Inf. Softw. Technol..

[8] Barricelli B.R., Casiraghi E., Fogli D. (2019). A Survey on Digital Twin: Definitions, Characteristics, Applications, and Design Implications. IEEE Access.

[9] Zhuang J., Chen Y., Chen X. (2018). A new simplified modeling method for model predictive control in a medium-sized commercial building: A case study. Build. Environ..

[10] Lewis P.R., Platzner M., Rinner B., Tørresen J., Yao X. (2016). Self-Aware Computing Systems.

[11] Minku L.L., Esterle L., Nebehay G., Chen R. (2016). Knowledge representation and modelling: Structures and trade-offs. Self-Aware Computing Systems: An Engineering Approach.

[12] Kounev S., Lewis P., Bellman K.L., Bencomo N., Camara J., Diaconescu A., Esterle L., Geihs K., Giese H., Götz S. (2017). The notion of self-aware computing. Self-Aware Computing Systems.

[13] Ab Wahab M.N., Nefti-Meziani S., Atyabi A. (2015). A comprehensive review of swarm optimization algorithms. PLoS ONE.

[14] Gendreau M., Potvin J.Y. (2010). Handbook of Metaheuristics.

[15] Kennedy J., Eberhart R. Particle swarm optimization. Proceedings of the ICNN’95—International Conference on Neural Networks.

[16] Zhang N., Bahsoon R., Theodoropoulos G. (2020). Towards engineering cognitive Digital Twins with self-awareness. Proceedings of the 2020 IEEE International Conference on Systems, Man, and Cybernetics (SMC).

[17] Ljung L., Kailath T. (1987). System Identification: Theory for the User.

[18] Clerc M., Kennedy J. (2002). The particle swarm—Explosion, stability, and convergence in a multidimensional complex space. IEEE Trans. Evol. Comput..

[19] Phanden R.K., Sharma P., Dubey A. (2021). A review on simulation in Digital Twin for aerospace, manufacturing and robotics. Mater. Today Proc..

[20] Li L., Aslam S., Wileman A., Perinpanayagam S. (2022). Digital Twin in Aerospace Industry: A Gentle Introduction. IEEE Access.

[21] Tao F., Zhang H., Liu A., Nee A.Y.C. (2019). Digital Twin in Industry: State-of-the-Art. IEEE Trans. Ind. Inform..

[22] Melesse T.Y., Di Pasquale V., Riemma S. (2021). Digital Twin models in industrial operations: State-of-the-art and future research directions. IET Collab. Intell. Manuf..

[23] Sarantinoudis N., Tsinarakis G., Dedousis P., Arampatzis G. (2023). Model-Based Simulation Framework for Digital Twins in the Process Industry. IEEE Access.

[24] He Q., Wang L., Liu B. (2007). Parameter estimation for chaotic systems by particle swarm optimization. Chaos Solitons Fractals.

[25] Schwaab M., Biscaia E.C., Monteiro J.L., Pinto J.C. (2008). Nonlinear parameter estimation through particle swarm optimization. Chem. Eng. Sci..

[26] Zha F., Sheng W., Guo W., Qiu S., Deng J., Wang X. (2019). Dynamic Parameter Identification of a Lower Extremity Exoskeleton Using RLS-PSO. Appl. Sci..

[27] Polsena A., Kongjeen Y., Watcharakhup S. Identifying Parameter and PI Tuning of DC Motor for Predict Behavior based on PSO. Proceedings of the 2021 9th International Electrical Engineering Congress (iEECON).

[28] Gupta J., Hussain A., Singla M.K., Nijhawan P., Haider W., Kotb H., AboRas K.M. (2023). Parameter Estimation of Different Photovoltaic Models Using Hybrid Particle Swarm Optimization and Gravitational Search Algorithm. Appl. Sci..

[29] Qaraad M., Amjad S., Hussein N.K., Farag M., Mirjalili S., Elhosseini M.A. (2024). Quadratic interpolation and a new local search approach to improve particle swarm optimization: Solar photovoltaic parameter estimation. Expert Syst. Appl..

[30] Deng F., Wang J., Wang J. (2023). Estimation of a five-parameter JONSWAP spectra with an improved particle swarm optimization. Appl. Ocean Res..

[31] Brunton S.L., Proctor J.L., Kutz J.N. (2016). Discovering governing equations from data by sparse identification of nonlinear dynamical systems. Proc. Natl. Acad. Sci. USA.

[32] Deng X. System Identification Based on Particle Swarm Optimization Algorithm. Proceedings of the 2009 International Conference on Computational Intelligence and Security.

[33] Tao F., Cheng J., Qi Q., Zhang M., Zhang H., Sui F. (2018). Digital twin-driven product design, manufacturing and service with big data. Int. J. Adv. Manuf. Technol..

[34] Brylina O.G., Kuzmina N.N., Osintsev K.V. Modeling as the Foundation of Digital Twins. Proceedings of the 2020 Global Smart Industry Conference (GloSIC).

[35] Liu X.Q., Zhang H.Y., Liu J., Yang J. (2000). Fault detection and diagnosis of permanent-magnet DC motor based on parameter estimation and neural network. IEEE Trans. Ind. Electron..

[36] Medeiros R.L., Lima Filho A.C., Ramos J.G.G., Nascimento T.P., Brito A.V. (2018). A novel approach for speed and failure detection in brushless DC motors based on chaos. IEEE Trans. Ind. Electron..

[37] Rahmat M. (2009). Application of self-tuning fuzzy PID controller on industrial hydraulic actuator using system identification approach. Int. J. Smart Sens. Intell. Syst..

[38] Tony Thomas A., Parameshwaran R., Sathiyavathi S., Vimala Starbino A. (2022). Improved position tracking performance of electro hydraulic actuator using PID and sliding mode controller. IETE J. Res..

[39] Elias N., Yahya N. (2018). Simulation study for controlling direct current motor position utilising fuzzy logic controller. Int. J. Automot. Mech. Eng..

[40] Ponce P., Rosales J.A., Molina A., Ponce H., MacCleery B. (2020). Designing a robust controller using SMC and fuzzy artificial organic networks for brushed DC motors. Energies.

[41] Premkumar K., Manikandan B. (2014). Adaptive Neuro-Fuzzy Inference System based speed controller for brushless DC motor. Neurocomputing.

[42] Barasuol V., Villarreal-Magaña O.A., Sangiah D., Frigerio M., Baker M., Morgan R., Medrano-Cerda G.A., Caldwell D.G., Semini C. (2018). Highly-integrated hydraulic smart actuators and smart manifolds for high-bandwidth force control. Front. Robot. AI.

[43] Lovrec D., Kastrevc M., Ulaga S. (2009). Electro-hydraulic load sensing with a speed-controlled hydraulic supply system on forming-machines. Int. J. Adv. Manuf. Technol..

[44] Krishnan R. (2001). Electric Motor Drives: Modeling, Analysis, and Control.

[45] Manring N. (2005). Hydraulic Control Systems.

[46] Fazdi M.F., Hsueh P.W. (2023). Parameters Identification of a Permanent Magnet DC Motor: A Review. Electronics.

[47] Wonohadidjojo D., Kothapalli G., Hassan M. (2013). Position Control of Electro-hydraulic Actuator System Using Fuzzy Logic Controller Optimized by Particle Swarm Optimization. Int. J. Autom. Comput..

[48] Wargantiwar N., Rambhad K., Ballamwar P. (2018). Hydraulic Systems and Hydraulic Leakages—A Review. Int. J. Anal. Exp. Finite Elem. Anal. (IJAEFEA).

[49] Blank J., Deb K. (2020). Pymoo: Multi-Objective Optimization in Python. IEEE Access.

[50] Jokinen A., Calonius O., Pietola M., Gorle J. (2019). Effects of oil contamination level, flow rate and viscosity on pressure drop development and dirt holding capacity of hydraulic filter. Proceedings of the Fluid Power Systems Technology.

